# Fraud triangle and earnings management based on the modified M-score: A study on manufacturing company in Indonesia

**DOI:** 10.1016/j.heliyon.2023.e13649

**Published:** 2023-02-11

**Authors:** Niluh Putu Dian Rosalina Handayani Narsa, Lesta Mega Evi Afifa, Oktaviani Ari Wardhaningrum

**Affiliations:** a Department of Accounting, Faculty of Economics and Business, Universitas Airlangga, Jl. Airlangga 4-6, Surabaya, Indonesia; b Department of Accounting, Faculty of Economics and Business, Universitas Jember, Jl. Kalimantan Tegalboto 37, Jember, Indonesia

**Keywords:** The modified Beneish M-score, Earnings management, Fraud triangle, Indonesia, Manufacturing company

## Abstract

This study combines the concept of a fraud triangle and a modified Beneish M-score to detect factors that trigger earnings management. The modified M-score formula used in this study contains five original and four additional ratios. A sample of 284 manufacturing companies listed on the Indonesia Stock Exchange in the period 2017–2019 is used. Based on the logistic regression test and *t*-test, the results show that asset growth, changes in receivables to sales, and auditor changes have a negative relationship and debt ratio has a positive relationship with earnings management. In addition, return on assets has no relationship with earnings management. In other words, manipulator firms are subject to greater pressure on leverage and have fewer independent commissioners. This study is the first to utilise the modified Beneish M-score model to detect earnings management in manufacturing companies in Indonesia. The effectiveness of this model makes it a valuable tool in detecting fraud and is expected to be useful for future research.

## 1 Introduction

In carrying out the task of preparing financial statements, management is often motivated to manipulate those statements, obtain higher share prices, demonstrate compliance with financing agreements, meet company projections and investor expectations, and obtain more favourable financing or financing terms [67]. It is thus not uncommon for management to be engaged in fraudulent activity. Fraud in relation to financial statements also occurs in almost all companies in the world, including Indonesia; no entity is immune to the threat of fraud [2]. As a country that still has a high corruption index – evidence that opportunistic behavior is still commonplace here – we can straightforwardly find numerous fraudulent financial statement cases in Indonesia, for example PT KAI (railroad company), Kimia Farma (pharmaceutical industry company), Bank Lippo (banking company), PT Hanson International (property company) and PT Garuda Indonesia (airlines company). Based on the results of the Report to the Nations [14] survey by the Association of Certified Fraud Examiners (ACFE), 10% of financial statements for the period examined were fraudulent. ACFE Indonesia [9] identified 239 cases of fraud in the country, with financial statement fraud accounting for 6.7% or 16 cases. In relation to the results for the media, 93 respondents or 38.9%, stated that the financial media contributed the most to disclosing fraud in Indonesia; thus, exploring earnings management in Indonesia will always be an interesting issue.

[50] note that the three elements of the fraud triangle theory reliably describe the triggers for the manipulation of financial statements. Similarly [7,70,82], and [35] argue that manipulation occurs because of pressure from within the company and responsibility to external parties. Although various studies argue that each element of the fraud triangle has a relationship to financial statement fraud, there are several contradictory studies [82]. uses data from the United States Securities and Exchange Commission Electronic Data Gathering, Analysis, and Retrieval system to show that financial statement fraud only has a significant positive relationship with the elements of pressure and opportunity; there is no relationship with the rationalisation element. This opinion is supported by the results in Refs. [4,76]. However [56], uses data from mining companies listed on the Indonesia Stock Exchange (IDX) and finds a relationship between financial statement fraud and the fraud triangle elements of pressure and rationalisation, but not with the element of opportunity. This finding is supported by the finding of [7] that rationalisation has a relationship with financial statement fraud. Some of the differences between the various studies stem from differences in the data sources used; each study takes data from different agencies and uses different settings resulting in inconsistencies. This study, therefore, measures the fraud triangle using six proxies in total.

Then, those six proxies of fraud triangle will be associated with earnings management, but the problem is, as stated by Ref. [67], he argues that researchers do not always have a good ability to measure earnings management. An appropriate and accurate model is needed to do so. Among earnings management measurement models, the Beneish M-score is considered the most appropriate tool for detecting financial statement fraud in single companies [ [59,88]] [67]. states that of the existing forensic tools, the Beneish M-score is cost-effective and more accurate at detection than others [52]. states that the Beneish M-score is more accurate than the Altman Z-score in detecting Enron Corporation manipulation. In addition [24], also states that the predictive ability of the Beneish M-score is stronger than the CFEBT Model.

Although the M-score is commonly used to measure fraud, there are several studies [ [1,8,18,26,66,83], and [86]] stating that the M-score can also be used to detect so-called black earnings management that occurs in companies [66]. also mention that companies that manipulate earnings tend to mislead stakeholders, violate rules, and are destructive (or referred to as black). In addition to confirming that earnings management coincides with earnings manipulation, which is an aspect of fraud, this research examines whether the fraud triangle components and M-score can reveal the phenomenon of earnings manipulation within companies.

Over time, modifications of the Beneish model have been required. The Beneish M-score was developed around 1990. There are several studies using this model for detecting earnings manipulation in Indonesia – for example, recent research by Ref. [28] investigates companies listed on Kompas 100 [39], research manufacturing companies, and [30] explore the non-financial sector. Those three studies used the original Beneish M-score model [12]. Nevertheless, the accounting standards and financial reporting disclosure rules of the United States, on which the model for those studies is constructed, differ from those of Indonesia. Therefore, it is necessary to modify the Beneish model so that each ratio accurately and effectively defines profit manipulation.

The aim of this study is to test the relationship between components of the fraud triangle and earnings management measured using the Beneish M-score model. This study adds four ratios in the Beneish M-score formula based on the modifications of [51] for China. One of the components of this modified Beneish M-score lies on the utlization of fixed asset ratio that assumed to be suitable because in Indonesia, asset fraud accounts for 28.9% of occupational fraud [9]. The modification we conduct to the Beneish M-score formula in is the novelty in this research, which to the best of author's knowledge there has never yet succesfully accomplished in earnings management research in Indonesia. This study uses companies engaged in the manufacturing sector listed on the IDX for the period 2017–2019. There are high levels of financial statement fraud in Indonesia across various business sectors, including manufacturing. The Ministry of Industry [42] in a press statement by then minister Airlangga Hartarto, states that the manufacturing industry contributes 20% to GDP, 30% to taxation revenues, and 74% to exports. According to the [3], the manufacturing sector has a fairly high level of vulnerability to manipulation. The observations are selected for the period 2017 to 2019 for several reasons. According to the Central Statistics Agency in Indonesia, the manufacturing sector's growth has weakened since the second quarter of 2017. Although it strengthened briefly in the third quarter of 2017, the sector continued to weaken until 2019 (Adharsyah, 2019). As stated by Refs. [23,61], and [81], when the financial performance of the company is decerase, it will encourage the company to engage financial fraud, thus, it would be interesting to conduct research on this matter. Furthermore, we made 2019 as our cut-off year considering that a rapid market-wide financial crisis that was caused by an impending global health pandemic began in the first few months in 2020. As already documented by several researchers [ [11,22,68]] in which they concluded that the presence of this pandemic has a noteworthy effect on the company's financial performance, thus it is prudent to exclude the year to avoid the noise results.

The results of the statistical tests on a sample of 284 manufacturing companies listed on the IDX for the period 2017–2019 show fraud in 50.4% of cases using the modified Beneish M-score calculation; this calculation is based on the results of recent research by Ref. [50]. In addition, the AGROW ratio has a positive relationship, and the IND ratio has a negative relationship with earnings management. The main contribution of this study is that it is, to the best of our knowledge, the first Indonesian study to use a modified Beneish M-score as a measure of earnings management and to link that with the fraud triangle components using six proxies. This research also contributes to the development of the theory and practice of detecting fraudulent financial statements in Indonesia and may be useful as a reference for future research, especially in its development of the Beneish M-score formula and proxies to describe each element in the fraud triangle concept.

This paper next presents a literature review. Following the review of previous research, we develop hypotheses and conceptual frameworks. In the next section, we discuss the research methodology and results and, finally, close with conclusions and suggestions.

## 2 Literature review and hypotheses development

### 2.1 Agency and signalling theory

Agency theory explains the underlying and inevitable problem of conflict of interest in agreements between an agent and the principal [40]. The theory addresses the relationship between management and third parties whose interests in a company differ. In this case, the divergence results from management engaging in illegal efforts, such as falsifying data on income and expenses incurred, to fulfil the interests of third parties.

Signal theory addresses information asymmetry in respect of data quality and quantity between parties internal and external to the company [79]. In developing signal theory [69], describes the importance of external party control over the reporting of a company's financial statements. Because management has better information, information or signals must be properly conveyed to external parties so that there is no information gap. Such a gap creates uncertainty and negatively impacts a company's image [36]. Signal theory emphasises the importance of information released by management because it has an impact on the investment decisions of external parties and is responsible for maintaining the good image a company has built. These two theories can explain how earnings management is manipulated through the fraud triangle component with the ratios Asset Growth (AGROW), Debt Ratio (DR), Return on Assets (ROA), Nature of Industry (PP), Affectivity of Supervisor (IND), and Auditor Changes (AUDCHANGES).

### 2.2 Fraud triangle and earnings management

Clinard and Cressey [17] identify the concept of the fraud triangle to explain the factors that trigger fraud in a company. The fraud triangle theory is considered the most robust among theories explaining the factors that trigger fraud [30]. find that the fraud triangle theory is well developed to understand the motivation for fraud inside a company.

Pressure is viewed as the most powerful trigger for fraud [35] and can come from both internal and external factors. Internal factors take the form of demands made on employees to achieve particular financial targets and financial stability by the company. Financial stability is assessed through asset growth. A good rate of asset growth will lead to the assumption that the company is able to utilise its assets well in increasing the current year's profit [30]. In addition to pressure related to financial stability, third parties may create pressure in the form of high financial targets and low debt levels. In other words, third parties want the company to attain maximum profits in the year with minimum expenses and debt [60]. explains how financial targets and leverage affect the level of fraud in the company through return on assets and debt ratios. There are various reasons that companies want low leverage, including convenience when applying for loans from third parties (banks) to maximise production. An increase in the proportion of debt not matched by an increase in asset utilisation may indicate manipulation [63]. In addition, according to Ref. [63], when the company's leverage and credit risk are relatively high, there is a high possibility of the company violating credit agreements. Therefore, leverage is appropriate as an indicator to detect manipulation [43]. Financial targets are equally suitable as indicators of the manipulation that occurs in the company. When the company wants to meet the financial targets set by stakeholders, it may record fictitious income [17].

The next component in the fraud triangle is opportunity. Cheating will not occur if the situation is not conducive to that behavior [53]. Weak internal controls provide opportunities for perpetrators to take actions that are detrimental to the company. The statement on auditing standards, SAS No. 99 [6], sets out several conditions related to the element of opportunity in the practice of manipulating financial statements. These conditions include the nature of the industry, as proxied by changes in receivables on sales (PP) and the effectiveness of supervision proxied by commissioner's independence (IND). Sometimes, in practice, management actually spearheads the manipulation and asks for help from other employees. It is often the case that financial statements containing manipulation have entailed management controls that appear to outsiders to be effective but are flawed when explored more deeply. Management overrides of controls can occur in unexpected ways. The proxy for measuring the nature of the industry is taken from Ref. [60] and the proxy for measuring the effectiveness of supervision is taken from research by Refs. [30,60].

The last component in the fraud triangle concept is rationalisation. Rationalisation can be defined as a person's attitude or character that justifies fraudulent practices [77]. The perpetrator will try to find any reason to justify their actions. For example, manipulative actors rationalise their actions by borrowing company money so that they do not harm any party [75].

Clinard and Cressey [17] explain how a person performs a manipulation that is motivated by three components, namely pressure, opportunity and rationalisation [77]. stated that economic motives, both incentives and penalties, always arise inside companies, such as profit targets, growth maintenance, bonus cuts, poor performance evaluations, or even dismissals.

**Fig. 1:**
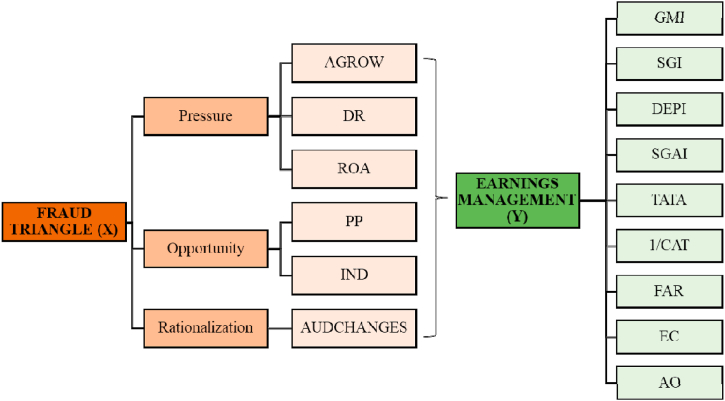
Conceptual framework.

According to Ref. [8], earnings management occurs when management intentionally uses considerations in the financial statements and the arrangement of existing transactions to change the results of the financial statements to mislead stakeholders about the company's performance [90]. state that earnings management can be divided into three types, namely white, grey, and black. In short, favourable earnings management (white) increases report transparency, destructive earnings management (black) involves misrepresentation and fraud, and grey earnings management is the manipulation of reports within the bounds of compliance with the bright-line standards, which can be either opportunistic or efficiency improvements. In this study, all the companies studied are considered to engage in earnings management with a detrimental impact (black earnings management). Fig. 1 potraying our conceptual framework. We combine the concept of fraud triangle (using the original work from Ref. [17]) with earnings management (using the Modified Beneish M-Score model by Ref. [51]).

### 2.3 The Modified Beneish M-score

Beneish [12] uses eight ratios to detect earnings management and classifies the results according to which companies commit financial statement fraud: the Day's Sales in Receivables Index (DSRI), Gross Margin Index (GMI), Asset Quality Index (AQI), Sales Growth Index (SGI), Depreciation Index (DEPI), Sales Growth General and Administrative Expenses Index (SGAI), Leverage Index (LVGI), and Total Accruals to Total Assets (TATA). However, this study does not use DSRI, AQI, and LVGI because, according to Ref. [51], the three ratios have a relationship with earnings management, so they cannot be included in the modified M-score formula.

The research conducted by Beneish is based on the accounting standards and policies of the United States in 1999. Beneish's own formula has been widely used and tested, and the results vary. Modifications are needed to update this formula and increase its accuracy [51]. In this study, four new ratios are included derived from the research of [51]. These will later become the basis for grouping the companies as being manipulators (performing earnings management) or not. The ratios added are 1/CAT, FAR, EC, and AO. Some recent research exploring earnings management also uses the Beneish M-score to detect the financial statement fraud [ [1,8,18,26,30,45,55,66,71,83]]. They present a strong basic argument that the Beneish M-score is more effective in predicting ‘black’ earnings management.

### 2.4 Hypotheses development

#### 2.4.1 Financial Stability's relation to earnings management

According to SAS No. 99 [6], pressure on management comes from outside the company and takes the form of needing to meet the demands and targets of third parties and having responsibility for outcomes. Consistent earnings are demanded [44]. Management that manipulates earnings faces greater pressure in respect of the company's financial stability, leverage, and financial targets [30]. Such pressure is usually generated by stakeholder demands that do not consider the actual limits of the company. This is explained in agency theory [40] as one of the things that reveal the divergence of interests between parties in a company.

Differ with banking sector where financial stability can be achieved when the movement of the Financial System Stability Index is maintained in the normal zone, in the manufacturing sector, financial stability is a condition when the company looks fine and is able to compete with other companies so as to provide satisfaction to shareholders [38]. From this, indubitably, the company is indirectly required to create a stable financial condition. This encourages companies to commit fraudulent financial statements in order to produce good financial conditions and meet established stability standards.

Financial stability will certainly lead to growth in company assets [6], thus, company financial stability can be reflected using AGROW because the instability of a company's performance can be understood in various ways; it may, for example, be seen as reflecting the way the company utilises its assets [ [30,53,60]). The inability of management to maximise assets causes changes in assets over a certain period to be too high or too low [34]. When the company has stable growth, investors and creditors will be encouraged to invest their funds. In addition, the company's image will improve when it is stable [74]. find that the company's image could be improved through environmental responsibility efforts while simultaneously practising earnings management. Similar [87], stated found the positive correlation between bank growth with earnings management. They stated that if the companys' assets are high, then the companies tend to be more risk taker so that they are more prone in engaging to earnings management activities. The similar idea also stated by Ref. [15]. In general, companies with high asset growth really depend on capital reinforcements, which lead them to earnings management activities.

The utilisation of assets is clearly illustrated in the financial statements. However, because assets are within reach of management, their reflection in the financial statements is easily manipulated [69]. emphasises that signal theory, according to Ref. [79], is concerned with addressing an information gap. Management can easily manipulate the values of the company's assets and expenses in order to meet financial targets. [26,48], and [76] state that companies with stable finances tend to be involved in earnings management or in other words companies with fraud receive more pressure on financial stability, thus, we formulate our first hypotehesis as follow:

H1 Financial stability has a positive relationship with earnings management

#### 2.4.2 Leverage's relation to earnings management

According to Ref. [78], management faces various pressures, including obtaining additional debt or external financing to ensure the company's competitiveness and status as a going concern. In detecting earnings manipulation, external pressure is proxied using a leverage ratio, namely the ratio between total liabilities with the total assets. According to Ref. [43], leverage is an indicator of earnings management when debt covenant violations occur.

High leverage indicates the company has a large debt and high credit risk. The relatively high credit risk will trigger a reduction in creditor confidence in lending to the company [55]. In a similar vein, according to Ref. [32], when the company's leverage and credit risk are relatively high, there is also a high possibility of the company violating the credit agreement.

Beneish [12] states that, in accordance with agency theory, management will manipulate debt so that the company is attractive to investors. It is also easier for companies to obtain additional funding from creditors to facilitate operational activities. According to signal theory [69], the disclosure of corporate debt levels to stakeholders should be as transparent as possible. If the disclosure signal is not optimal, it will result in bias in decision making. [30,84], and [60]state that companies that have high leverage tend to be involved in earnings management. Thus, we formulate the following hypothesis:

H2 Leverage has a positive relationship with earnings management

#### 2.4.3 Financial Target's relation to earnings management

Financial target is the situation whereas the company determining the financial goals – may be absolute or relative – at a certain period which then normally will be compared with other period, units, or entities [78]. Even tough the financial target can be expressed in many terms, such as operating income, earnings per share, incremental net revenue, EBIT, EBITDA, return on equity, cash flow from operating activities, and so forth, but return on assets (ROA) is considered as the most appropriate and common measure in conveying the financial target, as it also suggested by SAS No. 99 [6]. SAS No. 99 [6] explains that the relationship between the desire for financial statement performance and external pressures (such as that from investors, competition, and economic conditions) encourages management to manipulate earnings. Together with accounts receivable, sales accounts – which is part of the calculation of ROA – are vulnerable to manipulation. In a similar vein [35], also argue that financial targets trigger management to manipulate financial statements.

[77] state that to assess the performance of management through sales growth and the provision of bonuses, we can use the ROA. The trick is to compare the ROA of a particular year with the ROA of the previous year. Usually, when the ROA target for a year has not been met, the management will perform some manipulation on the sales posting to meet sales targets determined by external parties.

The misalignment of interests, described by Ref. [40] in agency theory, results in management's feeling compelled to manipulate records to fulfil the interests of outsiders and earn bonuses without regard for the impact on the company's image. Signal theory requires that financial statements (signals) are presented in accordance with the state of the company to satisfy investors and avoid future adverse effects [ [69,79]] [30,56]. find that companies with high sales targets tend to be involved in earnings management. In a study focusing on the scandal involving financial statement fraud, Toshiba [21], find that pressure (using ROA as a proxy) has a strong relationship with financial statement fraud (using the Beneish M-score as a proxy), so as with [30,60]. Based on this finding, we propose the following hypothesis:

H3 Financial target has a positive relationship with earnings management

#### 2.4.4 Relationship between nature of the industry and earnings management

SAS No.99 [6] states that the nature of the industry is a risk that arises in a company when its operational activities are based on estimates. Companies that use a lot of estimates in their operations are particularly vulnerable to fraud. In addition to errors due to estimation, manipulation is usually also carried out in conjunction with efforts to reduce costs related to receivables and sales.

Benish [12] explains that the costs associated with sales are often manipulated to demonstrate to investors that the company's performance in generating profitability is very good. In addition, manipulation is carried out with the aim that management gets a bonus for its performance [17]. Selling costs alone include salaries, advertising costs, manufacturing costs, rent, and all costs and taxes directly related to the production and sale of the product. In this case, the use of power and good signals [ [69,79]] make it easier for the perpetrator to manipulate. The calculation of the cost of sales is usually based on existing documents such as purchase orders and invoices. However, these documents can be manipulated by the authorities. SAS No. 99 [6] states that if a bad environment exists in a company, then employees will be ready to help their superiors carry out manipulations such as authorising sales documents which will later generate fictitious income in financial statements.

SAS No. 99 [6,64] state that in calculating the nature of the industry, one of the indicators that can be used is the change in accounts receivable on sales [60,76]. state that the nature of the industry, using changes in receivables on sales as a proxy, has a relationship with earnings management. Similarly [71], using the Beneish M-score as the proxy for earnings management, finds empirical evidence of earnings management through a disproportionate rise in receivables. Therefore, the researcher formulates the following hypothesis:

H4 The nature of the industry has a positive relationship with earnings management

#### 2.4.5 Relationship between effectiveness of supervision to earnings management

In SAS No. 99 [6], effective supervision is the availability of an adequate supervisory unit to monitor the company's business activities. The manager's capability is also important to manipulate because he has the power and free access to the company's assets. This usually occurs because of the weak internal control of the company's board of commissioners and audit committee, as well as extensive management power.

According to the agency theory of [40], management, as an agent, has access to company information that is more detailed, accurate and complete than that available to the principal. The knowledge of investors and other outside parties concerning the company's assets will not be as detailed as that of management. Information asymmetry [69] usually presents management with the opportunity to manipulate. The capabilities of management will trigger manipulation opportunities for the fulfilment of financial targets.

SAS No. 99 [6,77] stated that in calculating the effectiveness of supervision, one of the indicators that can be used is independent commissioners. Managers who intend to manipulate reporting tend to reduce the independence of the number of commissioners. Therefore, the effectiveness of supervision is measured by the ratio of independent commissioners to the number of the company's Board of Commissioners (IND) [30,55,58]. state that companies that have independent commissioners tend to be involved in the manipulation of financial statements. Hereafter, we propose the hypothesis:

H5 Effectiveness of supervision has a negative relationship with earnings management

#### 2.4.6 The relationship between auditor changes and earnings management

The going concern principle is the assumption by management and external parties that the company intends to operate in the long term. An opinion that the company is a going concern is usually issued by the auditor. This suggests that the auditor has a role in assessing the company's ability to manage its assets and generate profit. Companies that are not able to generate profits as targeted by third parties tend to manipulate [12]. supports this view, stating that companies with weaker profitability tend to engage in earnings management practices.

Management tends to change auditors frequently to avoid detection [20]. Auditor independence is, in terms of signal theory, expected to produce a reliable audit opinion so that it can serve as a signal to stakeholders that the financial statements presented by management reflect the company's real conditions [69]. The relationship between management and auditors is management rationalisation. Auditor observations have an effect on audit risk and results, which are of concern to management. In this study, external auditors who experience changes serve as proxies for auditor changes. A change of auditors is intended to erase the company's history of fraud and offer a way for the company to cover up the manipulation of financial statements. Differences in parties' interests trigger management's desire to erase the history of fraud [40]. In.

SAS No. 99 [6] states that in an effort to determine audit changes of a company, a dummy variable can be used that takes the value of ‘1‘ for those that make auditor changes and zero otherwise. Managers who intend to manipulate reporting tend to change auditors frequently [60,76]. state that companies that frequently perform audit changes tend to be involved in the manipulation of financial statements. Another recent study by Ref. [21] finds that audit opinion has a strong relationship with financial statement fraud. Based on the description above, we formulate the following hypothesis:

H6 Audit changes have a positive relationship with earnings management.

## 3 Research method

### 3.1 Research data

Based on calculations using the purposive sampling method, we obtained a sample of 284 observations, as seen in Table 1. The type of data used is secondary data obtained from financial and annual reports of companies in the manufacturing sector listed on the IDX for the years 2017–2019. We utilise various websites to complete our data: 1) www.sahamok.net for a list of companies in the manufacturing sector; 2) www.idx.co.id for financial reports and annual reports of companies in the manufacturing sector listed on the IDX; 3) https://m.id.investing.com for financial reports and annual reports not available on the official IDX website.

**Table 1:** Research sample.

Criteria	Year		
	2017	2018	2019
Manufacturing sector companies listed on the IDX in 2017–2019	157	167	182
Companies whose data are incomplete for research purposes	(37)	(42)	(47)
Companies that do not present financial statements in Rupiah currency	(31)	(32)	(33)
Number of samples that meet the criteria	89	93	102
Number of samples for 3 years of research	284		

### 3.2 Variable operational definition and variable measurement

#### 3.2.1 Earnings management

Earnings management is defined as the deliberate action by management of using judgement in financial statements and structuring existing transactions to change the results of financial statements so as to mislead stakeholders about the performance of a company and/or affect the outcome of contracts that depend on the accounting numbers reported by the company [8].

In explaining earnings management, the original Beneish M-score are used to group companies as fraudulent (manipulator) or not, using eight ratios that reveal unusual activities in the company's financial statements. However, to improve the accuracy of this formula and update previous research, we add four new ratios from Ref. [51]. The estimations are also based on the M-score model modified in Ref. [51] as more suitable for the Chinese market; this model is also suitable for Indonesia. For instance, the 1/CAT and FAR ratios are used because manipulation of assets in financial statements is the most common fraud of this type in the global context and the second most common in Indonesia [9]. Therefore, it is important to test whether the company's assets are manipulated. The remaining two ratios, EC and AO, are non-financial ratios for detecting earnings management, which is also quite an important factor. The five original ratios of the Beneish M-score model (GMI, SGI, DEPI, SGAI, and TATA) and four additional ratios are used to construct what is referred to as the Modified Beneish M-score. These nine ratios are used to classify manipulator and non-manipulator companies. Table 2 below presents nine variables used in this study as the modified Beneish M-Score. Another reason for using the Modified M-score is that in other methods, the focus of measurement is non-discretionary accrual with constant assumptions over time, and when the company does not meet the model criteria, it will result in type I or type II errors.

**Table 2:** Nine variables used in this study as the modified Beneish M-score.

No.	Ratio	Formula
1.	*Gross Margin Index* (GMI)	$\frac{{Gross\;Profit}_{t-1}/{Sales}_{t-1}}{{Gross\;Profit}_{t}/{Sales}_{t}}$
2.	*Sales Growth Index* (SGI)	$\frac{{Sales}_{t}}{{Sales}_{t-1}}$
3.	*Depreciation Index* (DEPI)	$\frac{\left[{Depreciation}_{t-1}/\left({PPE}_{t-1}+{Depreciation}_{t-1}\right)\right]}{\left[{Depreciation}_{t}/\left({PPE}_{t}+{Depreciation}_{t}\right)\right]}$
4.	*Sales and General Administration Expenses Index* (SGAI)	$\frac{{SGA}_{t}/{Sales}_{t}}{{SGA}_{t-1}/{Sales}_{t-1}}$
5.	*Total Accrual to Total Asset* (TATA)	$\frac{\left({Operating\;Income}_{t}-{Cash\;Flow\;From\;Operation}_{t}\right)}{{Total\;Assets}_{t}}$
6.	1/CAT (*Current Assets Turnover*)	$\frac{Average\;Current\;Assets}{Main\;Business\;Income}$
7.	*Fixed Assets Ratio* (FAR)	$\frac{Net\;Fixed\;Assets}{Total\;Assets}$
8.	*Equity Concentration* (EC)	1 = Total square of the percentage of five shareholders 0.5 0 = Total square of the percentage of five shareholders <0.5
9.	*Audit Opinion* (AO)	1 = unqualified opinion 2 = unqualified opinion with explanatory notes 3 = qualified opinion 4 = adverse opinion 5 = disclaimer opinion

The formula used is as follows:

M−value=−2,634+(0,009xGMI)+(0,043xSGI)+(0,067xDEPI)+(0,236xSGAI)−(2,191xTATA)−(0,11x1/CAT)−(0,253xFAR)−(1,869xEC)+(0,437xAO)

By terms [ [12,13]]:

For M-values > −1.78, a value of 1 is assigned (detected manipulator).

For M-value < −1.78, a value of 0 is assigned (detected non-manipulator).

### 3.3 Fraud triangle

The fraud triangle theory proposed by Ref. [17] explains an individual's decision to commit fraud. The theory sets out three factors that increase the likelihood of fraud, namely, pressure, opportunity and rationalisation. This study uses six ratios as proxies for the fraud triangle, which can be seen in Table 3.

**Table 3:** Independent variables.

Variable	Proxy	Indicator	Formula
Pressure	*Financial Stability*	AGROW	$\frac{{Total\;assets}_{t}-{Total\;assets}_{t-1}}{{Total\;assets}_{t}}$
	*Leverage*	DR	$\frac{Total\;Liability}{Total\;assets}$
	*Financial Target*	ROA	$\frac{{Earnings\;after\;tax}_{t-1}}{{Total\;assets}_{t}}$
Opportunity	*Nature of Industry*	PP	$\frac{{Receivable}_{t}-{Receivable}_{t-1}}{{Sales}_{t}-{Sales}_{t-1}}$
	*Effectiveness of Supervision*	IND	$\frac{Number\;of\;Independent\;Commissioners}{Total\;Board\;of\;Commissioners}$
Rationalisation	*Auditor Changes*	AUDCHANGES	0 = No change of auditor 1 = There has been a change of auditors

### 3.4 Data analysis technique

This study uses multiple logistic regression analysis to observe the effect of financial stability (AGROW), leverage (DR), financial targets (ROA), nature of the industry (PP), the effectiveness of supervision (IND), and auditor changes (AUDCHANGES) on earnings management in manufacturing sector companies listed on the Indonesia Stock Exchange for the period 2017–2019. Logistic regression is used because the nature of the dependent variable is categorical (manipulator vs non-manipulator). According to Ref. [47], logistic regression is useful to reduce the potential bias resulting from differences in the groups being compared. We did not perform normality or classical assumption tests on the independent variables; multiple logistic regression analysis does not require it [33]. Nevertheless, we test for multicollinearity and ensure that our independent variables are not correlated. In addition to using logistic regression to determine the relationship between variables, an additional *t*-test is performed to increase the confidence levels and ensure the results are robust. In the *t*-test, the sample is separated into two groups, namely manipulators and non-manipulators, and each of the fraud components is compared for the two groups. The statistical tests in this study are conducted using the IBM SPSS Statistics Viewer 25 software.

### 3.5 Empirical result

Table 4 presents the study's descriptive statistical data. Our hypotheses are tested using two types of testing. The first is logistic regression, and the second is a *t*-test that can be seen in Table 6, Table 7.

**Table 4:** Descriptive statistics.

	N	Minimum	Maximum	Mean	Std.
EM	284	0	1	0,50	0,501
AGROW	284	−5,697	0,921	0,04789	0,377113
DR	284	0,024	2,767	0,49514	0,328501
ROA	284	−77,311	33,641	0,06556	5,480859
PP	284	−0,375	0,527	0,04880	0,090899
IND	284	0,167	0,667	0,39345	0,090688
AUDCHANGE	284	0	1	0,15	0,362
Valid N (listwise)	284

**Table 5:** Pearson correlation results.

	AGROW	DR	ROA	PP	IND	AUDCHANGE	EM
AGROW	1.000	−0.003	0.019	0.062	0.049	−0.008	−0.139*
DR		1.000	−0.010	−0.221**	0.091	−0.027	0.233**
ROA			1.000	0.045	0.039	−0.129*	0.068
PP				1.000	−0.044	−0.082	−0.422**
IND					1.000	0.140*	−0.245**
AUDCHANGE						1.000	−0.256**
EM							1.000

*Correlation is significant at the 0.05.

**Correlation is significant at the level 0.01.

**Table 6:** Logistics regression coefficient test results.

		B	S.E.	Wald	df	Sig.	Exp(B)	95% C.I. for EXP(B)		VIF
								Lower	Upper	
Step 1	AGROW	−2.684	1.081	6.163	1	0,013	0.068	0.008	**0.568**	1.007
	DR	2.519	0.676	13.877	1	0,000	12.420	3.300	**46.752**	1.063
	ROA	0.051	0.044	1.378	1	0,240	1.053	0.966	**1.147**	1.022
	PP	−31.764	5.043	39.666	1	0,000	0.000	0.000	**0.000**	1.066
	IND	−10.643	2.161	24.247	1	0,000	0.000	0.000	**0.002**	1.036
	AUDCHANGES	−3.566	.628	32.213	1	0,000	0.028	0.008	**0.097**	1.048
	Constant	4.984	.958	27.069	1	0,000	146.054

**Table 7:** The *t*-test Results.

	EM	N	Mean	t	Sig.	Std. Deviation	Std. Error Mean
AGROW	Non-Manipulator	141	0.10072	2.363	0.019	0.148957	0.012544
	Manipulator	143	−0.00420			0.506011	0.042315
DR	Non-Manipulator	141	0.41811	−4.028	0.000	0.300609	0.025316
	Manipulator	143	0.57109			0.338041	0.028268
ROA	Non-Manipulator	141	−0.31153	−1.152	0.250	7.169582	0.603788
	Manipulator	143	0.43737			2.984754	0.249598
PP	Non-Manipulator	141	0.08740	7.826	0.000	0.096233	0.008104
	Manipulator	143	0.01074			0.066319	0.005546
IND	Non-Manipulator	141	0.41575	4.237	0.000	0.101659	0.008561
	Manipulator	143	0.37146			0.072253	0.006042
AUDCHANGE	Non-Manipulator	141	0.24823	4.448	0.000	0.433524	0.036509
	Manipulator	143	0.06294			0.243703	0.020379

Before testing the hypotheses, we conduct several tests to ensure that our statistical results are robust. The results of the feasibility test of the regression model returns a Chi-square value of 14.450 with a model significance value of 0.071. These results indicate that the regression model is acceptable. Based on the test of the overall model, the initial and final −2LogLikelihood (−2LL) values are compared; the initial value is 393.694, and the final −2LL value is 275.472. This shows that the regression model is fit and feasible for use in this study. Next, the coefficient of determination returns a Nagelkerke R Square value of 0.584. A classification matrix of 141 observations not indicated for earnings management indicates there are 110 that can be predicted by the logistic regression model. Furthermore, of the 143 observations indicated as engaged in earnings management, only 21.8% or 31 fail to match the predicted values. Therefore, it can be concluded that the regression model has a high predictive ability in identifying the sample that engages in earnings management, which is 78.2% of the 141 observations. Finally, the multicollinearity test indicates there is no correlation among our independent variables; this can be seen from the variance inflation factor below 10 [33].

Furthermore, we continue with pearson correlation test that provides initial idea about the correlationship of the variables tested in this research. Table 5 presents the pearson correlation results. The results are explained as follows: AGROW has a negative significant relationship with EM (r = −0.139; p = 0.019), DR has a positive significant relationship with EM (r = 0.233; p = 0.000), ROA has no significant relationship with EM (r = 0.068; p = 0.250), PP has a negaitive significant relationship with EM (r = −0.422; p = 0.000), IND has a negative significant relationship with EM (r = −0.245; p = 0.000), and lastly AUDCHANGES has negative significant relationship with EM (r = −0.256; p = 0.000).

The results of hypothesis testing are set out in Table 6, Table 7. The pearson correlation result as already mentioned above confirmed the results of the logistic regression and t-tests as it showed similar results. Taking into account all the statistical test that we have executed, it can be concluded that leverage, is the only independent variable that has a significant positive relationship with earnings management. This means that the higher the leverage of a company, the more likely it is that the company will engage in earnings manipulation. The effectiveness of supervision has a negative relationship with earnings management; the fewer independent commissioners on the board of commissioners, the more likely it is that the company will manipulate financial statements. Thus, H_2_ and H_5_ are accepted. Financial stability, the nature of the industry, and auditor changes have a significant negative relationship with earnings management. That is, the lower the value of each of these ratios, the more likely it is that the company manipulates its financial statements. However, because the direction of the relationship is opposite to that hypothesized, H_1_, H_3_, and H_6_ are rejected. The test results also show that H_4_ is rejected where the results when ROA serves as a proxy for the nature of the industry are insignificant; the higher the ROA, the more likely the company is to manipulate, but this variable cannot be used as a reference in assessing earnings management.

## 4 Discussion

### 4.1 Financial Stability's relation to earnings management

It is expected that company assets will be used effectively to produce maximum profits. Assets are a reflection of the company's wealth. Every company wants its asset to grow. This is because asset growth is one of the factors that determine the company's value. The more assets owned, the higher the value and the better the image of the company. The company will be attractive to investors, creditors, and other concerned parties. By contrast, if the company has growth below the industry average, then its value will be low. This will result in management being pressured by stakeholders, as explained by agency theory.

In contrast to our prediction, where we hypothesize that asset growth will be linked positively to earnings management, our results show a negative relationship (β = −2.685, p = 0.013). In other words, the higher the asset growth of a company, the lower its tendency to carry out financial fraud. The possible explanation maybe because when a company has a below-average asset growth, they will delay the cost of assets that are used proportionally to make financial statements look stable by manipulate the earnings [ [41,49]]. Therefore, a company with high asset growth, its financial statements can be declared stable so that the possibility of manipulation will be very small.

In a similar vein [35], mention that companies whose growth is below the industry average will thus encourage to manipulate profits. As well as [62] which also noted that the higher the asset value – which is the higher its role to company's growth – the lower management's tendency to engage in earnings management. That is might because the financial stability of the company merely due to management efficiency instead of management manipulation to gain a good impression from stakeholders. Stakeholders know whether the company is in good condition from the financial statements presented by management at the end of each accounting period. This is in line with signal theory's contention that financial statements are the best signal in conveying the state of the company. The result of this is supported by Ref. [78], who explain that poor financial stability and threats in the economy, industry, and context of a company indicate that management is under pressure, and this leads to manipulation of financial statements. Studies conducted by Refs. [1,41,62,65], and [72] also confirm this result, in which financial stability has a negative relationship with earnings manipulation.

### 4.2 The relationship of leverage to earnings management

The result of our statistical test for leverage (DR) is correspond to our hypothesis (β = −2.519, p = 0.000), where it is mean that a high increase in debt – that is not matched by an increase in profitability – indicates manipulation. The relatively high credit risk triggers a reduction in creditor confidence in lending to the company. The shareholders do not actually want the company to take on high debt to cover operational costs but rather want operational efficiency. Meanwhile, management tries to maximise operational activities for the sake of profitability. This divergence of interests is explained in agency theory and can lead to pressure from third parties. The pressure from shareholders triggers management to engage in manipulation.

If the company chooses to apply for a loan, the creditor will choose whether the rate of return on the company's debt is high or low. If a company has a high level of debt but its repayment ability is low, then its value will decrease. This consideration will be used as a benchmark for creditors in assessing whether or not the company can take on debt. By contrast, if the company has a low rate of return on debt, the company's profitability is also low. Management wishing to issue shares will try to improve the profitability reflected in the financial statements so that investors believe that the prospects of a company are good. The company's prospects can be seen from various factors in addition to profitability; one of these is the rate of debt repayment. In this study, manipulator companies tend to have a higher level of leverage than non-manipulator companies. Companies with high debt levels will pressure management to reflect the company's financial performance as capable of supporting its debt. Thus, it can be concluded that leverage measurement in an effort to detect fraudulent financial statements is very important. This is supported by the statement of [43], that leverage should be used as an indicator to detect earnings management actions when debt agreement violations occur. In the research by Refs. [30,65,85], and [60] it is also confirmed that companies with good leverage have indications of fraudulent financial reporting.

### 4.3 The relationship of financial target to earnings management

As can be drawn from Table 6, Table 7, it is reveal that our statistical test do not confirm with our third hypothesis (β = 0.051, p = 0.240) [77]. assess management performance by comparing the ROA of a particular year with that in the previous year. Usually, when the ROA target for a year has not been met, the management will engage in manipulation of sales posted. The sales account is crucial for the manufacturing sector because it is the largest source of income. Therefore, many companies are vying to increase their sales figures. However, based on our result, apparently the company's growth cannot be measured through profits and total assets alone, as also confirmed and supported by previous research from Refs. [53,54,58], and [10,27].

Based on agency theory, shareholders want management to ensure efficiency and effectiveness in various activities within the company, both operational and non-operational. It is aimed at maximising profit at a low cost. In addition, total assets alone cannot describe the company's overall growth. As also noted by Ref. [27], the company's growth cannot reflect all existing conditions unless it is followed by a decline in stock prices. From this statement, it can be concluded that the measurement of the company's growth in profitability through an assessment of ROA does not reflect the real situation. ROA cannot be used as a signal in detecting the condition of the company and is not correspond to signal theory. Correspondingly [62], also noted that managers do not deal with ROA as a financial target which is unfeasible to arrive at, thus it will discourage them to manipulate the earnings. In other words, it is possible that managers still assumes that the company's ROA target is considered attachable and normal, thus it does not trigger them to engage in fraudelent financial reporting.

### 4.4 The relationship of the nature of the industry to earnings management

Related to the rejection of our fourth hypotehesis, where we initially predicted that the relationship between nature of the industry with earnings management would be positive but it turned out to be negative (β = −31,674, p = 0.000), the possible explanation is because when the company is in ideal conditions (proxied by comparison of changes in receivables relative to changes in sales), there is minimal possibility that management will manipulate financial statements. In the other words, the lower the rate of change of receivables into sales, the higher the likelihood of management manipulating the financial statements. Even tough the lower rate of change of receivables into sales actually describes a good condition, but management still feels lacking and instead continues to engage in earnings management. Usually, manipulation occurs in accounts that estimate measurements, such as bad debt accounts, because it requires a subjective assessment [80].

However, this can be overcome when the company is able to formulate a good credit sales policy. This policy will allow the company to receive a refund of receivables collection according to the due period. This statement contradicts the agency theory, which holds that differences in the interests of parties lead to manipulation. In fact, in this study, the nature of the industry of non-manipulator companies is better than that of manipulator companies. This is a result of accommodation of interests between parties in the company allowing management to formulate the right receivables policy to avoid manipulation of financial statements. The result of this study is in line with the findings of [5,10,29] [54], and [57].

### 4.5 The relationship of effectiveness of supervision to earnings management

For the fifth hypothesis, regarding the relationship between affectivity of supervision and earnings management, this research confirmed the hypothesis as the direction of prediction that has been hypothesized same with the direction of the statistical result, which is a negative relationship (β = −3,566, p = 0.000). Independent commissioners are expected to provide guarantees for the supervision and internal control of a company. Manipulation is usually carried out by people who have more authority and free access within a company [89]. Independent commissioners are put in place with the aim of balancing the interests of management and shareholders. The existing divergence of interests can be minimised, according to Ref. [40], by appointing an independent commissioner.

The number of independent commissioners on the board clearly affects the level of control and supervision of a company. The greater the number of independent commissioners, the less likely the company is to manipulate financial statements [40]. use agency theory to argue that to increase the independence of the board of commissioners, the board must be dominated by parties from outside the company. Chtourou et al. [16] state that independent commissioners generally have better oversight of management, thus minimising the manipulation of financial statements. This statement supports signalling theory and the view that a board of commissioners is formed to maximise the delivery of signals to investors. This result is in line with the findings of [30,57,58,60], which state that the greater the number of independent commissioners, the lower the possibility of companies manipulating financial statements.

### 4.6 The relationship of auditor change to earnings management

Related to our last hypothesis, we predict that companies that frequently do the audit changes tend to be more involved in fraudelent financial statements, but based on the statistical result, this hypothesis was rejected as it is turn out that the relationship is negative (β = −3,566, p = 0.00). This study finds that companies that are detected as manipulators tend to change auditors less often than companies that are non-manipulators, or in other words, the more often auditor changes of the company, the lower the earnings management tendency. The possible reason is because the longer an auditor is in contact with management, there will be allegations of fraud in the audit results, particularly there is a possibility that the auditor cooperates with the management [50].

The audit opinion is a very important source of information for investors and requires an honest assessment, as explained in signal theory. Therefore, it is expected that the auditor will satisfy various parties. Considerations in selecting an auditor each year are based on the previous auditor's performance. The result of this study confirmed to what [31] stated. Management will rarely change auditors because of the personal relationship between the company and the auditor, causing a decrease in auditor independence. In addition, satisfaction with not detecting manipulation in the audit opinion is also one of the factors causing management to rarely change auditors.

Another factor is the period of cooperation. Long periods of cooperation can cause auditors to develop strong loyalty or emotional relationships with their clients, which can reach a stage where auditor independence is threatened. Long tenure also may result a decline in the quality of work and competence of the auditors when they begin to make unjustified assumptions instead of objectively evaluating current evidence. Dunn [25] states that the relationship between an auditor and his client can be close in one way or another regardless of the number of years of cooperation. In addition, the company can maximise the five-year cooperation period to cover up the manipulations. [37,50], and [73] also confirmed this negative relationship.

## 5 Conclusions, implications, limitations, and future research

As a forensic tool, the Beneish M-score is considered the most appropriate for detecting earnings manipulation in companies in developing countries and is also called the most appropriate tool to detect fraudulent financial statements. Although the M-score is commonly used to measure fraud, several studies [ [1,8,18,19,26,66,83,86]] state that the M-score could also be used to detect destructive or ‘black’ earnings management that occurs in companies [66]. also note that companies that manipulate earnings tend to manipulate stakeholders, violate the rules, and are destructive. In addition, earnings management intersects with earnings manipulation, which is part of fraud. Therefore, this study aimed to test whether the components of the fraud triangle and the modified Beneish M-scores can reveal the phenomenon of earnings manipulation in the company. The use of the modified Beneish M-score in this study is because the original Beneish M-score itself was built around 1990, and the context of calculation has changed. In addition, the accounting standards and financial reporting disclosure rules of the United States and Indonesia are different. Therefore, this study adds four ratios to the Beneish M-score formula, namely 1/CAT, FAR, EC, and AO, following [51] in China.

Being the first study that utilise the modified Beneish M-Score model to detect earnings management in Indonesia, this research indicates that from 284 manufacturing companies in Indonesia, 143 companies, or about 50.4% of the total sample, had an M-value of more than −1.78. This suggested that these companies tend to be manipulators. In addition, based on the results of the hypothesis testing using logistic regression reinforced by *t*-test, leverage (DR) had a positive relationship with earnings management, and affectivity of supervisor (IND), financial stability (AGROW), nature of industry (PP), and auditor changes (AUDCHANGES) had a negative relationship with earnings management, while financial target (ROA) was not found.

The practical implication of this research is that the external users of financial statements, especially auditors, investors, financial analysts, and various regulatory authorities, should maximise the use of the Beneish M-score as a cost-effective tool in detecting financial statement fraud, especially in the manufacturing industry. By paying attention to the modification of the Beneish M-score model, this research also has practical implications regarding for the use of Beneish M-scores in a manner more in line with Indonesian conditions. The research findings of this study can guide policy decisions making, especially regarding asset management, to mitigate financial statement fraud by the company. This is because the two additional variables included in the modified Beneish M-score (1/CAT and FAR) are related to fixed assets and have a relationship with the fraud triangle component used in this study. Other than that, related to our finding regarding the auditor changes, Indonesia's government need to consider the rules to oblige companies in rotating the auditor. Related to managerial and policy implication, the result of this study also benefits the internal stakeholders like managers and board of directors to formulate policy and strategies to deal with unwanted financial fraud, for instance like reward system, more prudent division of asset management tasks, uses the qualified auditor services, account receivable policy and improve the practice of corporate governance.

In addition to the relatively small number of observations and the limited years of observation in this study, another limitation is that the fraud triangle is used in viewing the earnings management phenomenon. In fact, fraud is not only triggered by the three elements reflected in the theory. The fraud triangle concept has been developed into a fraud hexagon, which will be an interesting concept to explore in further research. Future studies could modify the Beneish M-score in other ways to classify companies into manipulators and non−manipulators. This research also has weaknesses regarding the control variables. We did not use any control variables in order to focus our main independent variable for use in the new modified Beneish M-score model. Therefore, another opportunity for research is to utilise interesting control variables, like corporate governance. As noted by Ref. [46], in a country with high earnings management and weak investor protection, corporate governance could motivate managers to manipulate financial statement valuations. Lastly, next researchers can also utilise the Modified Beneish M-score model to detect the fraud in form of tax evasion.

## Author contribution statement

Niluh Putu Dian Rosalina Handayani Narsa: Conceived and designed the experiments; Analyzed and interpreted the data; Wrote the paper.

Lesta Mega Evi Afifa: Performed the experiments; Analyzed and interpreted the data; Contributed reagents, materials, analysis tools or data; Wrote the paper.

Oktaviani Ari Wardhaningrum: Contributed reagents, materials, analysis tools or data.

## Funding statement

This research did not receive any specific grant from funding agencies in the public, commercial, or not-for-profit sectors.

## Data availability statement

Data will be made available on request.

## Declaration of interest’s statement

The authors declare no conflict of interest.
